# Interdiffusion Behaviors and Microstructure Recombination Mechanisms of Fe_2_TiO_4_–CaO and FeTiO_3_–CaO Systems During Sintering at 1200 °C

**DOI:** 10.3390/ma18174091

**Published:** 2025-09-01

**Authors:** Bin Wang, Jianjun Gao, Feng Wang, Yue Yu, Yuanhong Qi

**Affiliations:** 1 State Key Laboratory for Advanced Iron and Steel Processes and Products, Central Iron and Steel Research Institute Co., Ltd., Beijing 100081, China; wang_bin919@126.com (B.W.); flywise@126.com (F.W.); 18622164233@163.com (Y.Y.); qiyh0525_cn@sina.com (Y.Q.); 2 Beijing Steel Research Institute of Hydrometallurgy Technology Co., Beijing 100081, China

**Keywords:** Vanadium–titanium magnetite, FeTiO_3_, Fe_2_TiO_4_, diffusion couples, interdiffusion coefficient

## Abstract

Vanadium–titanium magnetite (VTM) is an iron ore abundantly available in China. The dominant utilization route is blast furnace smelting; however, Ti in the ore deteriorates sinter strength, making it urgent to clarify Fe-Ti-Ca interactions during sintering. In this work, single-phase FeTiO_3_ and Fe_2_TiO_4_ were synthesized and each paired with CaO to fabricate diffusion couples. The couples were heated at 1200 °C for 30, 60, 90, and 120 min to investigate their interdiffusion behaviors and microstructure recombination mechanisms. The results show that, at 1200 °C, solid-state diffusion—not interfacial reaction—controls mass transfer in both FeTiO_3_-CaO and Fe_2_TiO_4_-CaO systems. Distinct Fe-rich and Ti-rich sublayers appear within the reaction zone, and banded CaTiO_3_ forms adjacent to the FeTiO_3_/Fe_2_TiO_4_ matrices. The interdiffusion coefficients were determined to be 4.08 × 10^−10^ cm^2^·s^−1^ and 7.81 × 10^−10^ cm^2^·s^−1^, and the growth of the reaction layer follows a parabolic law, which can be expressed as *x*^2^ = 2 × 1.562 × 10^−9^ *t* and *x*^2^ = 2 × 0.8159 × 10^−9^ *t*, respectively. The coefficients of determination exceed 0.90, indicating reliable regression fits.

## 1. Introduction

Vanadium–titanium magnetite (VTM) is an iron ore enriched in valuable metallic elements such as iron, titanium, and vanadium [1,2,3]. China holds abundant reserves of this resource [4]. As a critical raw material for multiple industries, it is classified as a strategic resource in many countries [5,6,7]. Developing VTM resources has alleviated China’s iron ore supply constraints while enabling efficient recovery of vanadium and titanium [8,9]. Vanadium derived from VTM is widely used in steelmaking [10], titanium alloys [11], catalysts [12], the chemical sector [13], energy storage [14], and hydrogen storage [15]. The titanium industry follows two principal value chains: a chemical route producing titanium dioxide pigment [16] and a nonferrous metallurgical route manufacturing titanium alloys for use in the chemical industry [17], aerospace [18], shipbuilding [19], and biomedical applications [20,21].

At present, the dominant mode of utilizing VTM in China remains blast furnace (BF) ironmaking [22], in which sinter is a key burden material [23,24]. The quality of the sinter strongly affects the BF’s technical and economic indices. Sinter produced from VTM generally exhibits lower strength than that from conventional iron ores [13]. Because VTM contains a higher titanium content than ordinary iron ores, it is both urgent and necessary to investigate Fe–Ti–Ca interactions during sintering. Prior studies have shown that TiO_2_ in VTM promotes the formation of hard and brittle perovskite (CaTiO_3_) while decreasing the amount of calcium ferrite, thereby exerting a pronounced influence on sinter quality [25,26,27,28]. Budzik [29] reported that suppressing the formation of calcium titanate effectively improves ore utilization in BF smelting. Ding [30] found that when the temperature exceeds the melting point of calcium ferrite, abundant calcium titanate forms in the CaO–Fe_2_O_3_–TiO_2_ system. Under continuous heating, the ore undergoes two reaction stages: calcium ferrite melts to form a liquid phase and TiO_2_ substitutes Fe_2_O_3_ in the liquid calcium ferrite to produce calcium titanate. Zhou [31] observed that the mineralogy of VTM sinter is complex—dominated by magnetite, hematite, calcium ferrite, and perovskite—and found that perovskite decreases sinter strength and the reduction degradation index (RDI).

It is well established that the presence of titanium—occurring predominantly as FeTiO_3_ and Fe_2_TiO_4_—is a principal cause of the deterioration in the sintering performance of VTM. Prior studies have investigated reactions during sintering, including those between fluxes and VTM [32], between calcium ferrite and calcium titanate [33], and between Fe_2_O_3_ and TiO_2_ [34]. However, existing research has paid limited attention to the CaO–FeTiO_3_/Fe_2_TiO_4_ systems, and the interactions between them remain largely unexplored. Accordingly, this study focuses on the FeTiO_3_-CaO and Fe_2_TiO_4_-CaO systems pertinent to VTM sintering, which provides a direct means to elucidate the mutual interactions among Fe, Ti, and Ca during the sintering process. Clarifying the diffusion mechanisms—as well as the characteristics and parameters of elemental diffusion—will lay the foundation for controlling elemental transport and reaction pathways; it will also help regulate the relevant reactions so that they proceed at rational, production-favorable rates [35].

## 2. Experimental

### 2.1. Synthesis of FeTiO_3_ and Fe_2_TiO_4_

**Figure 1:**
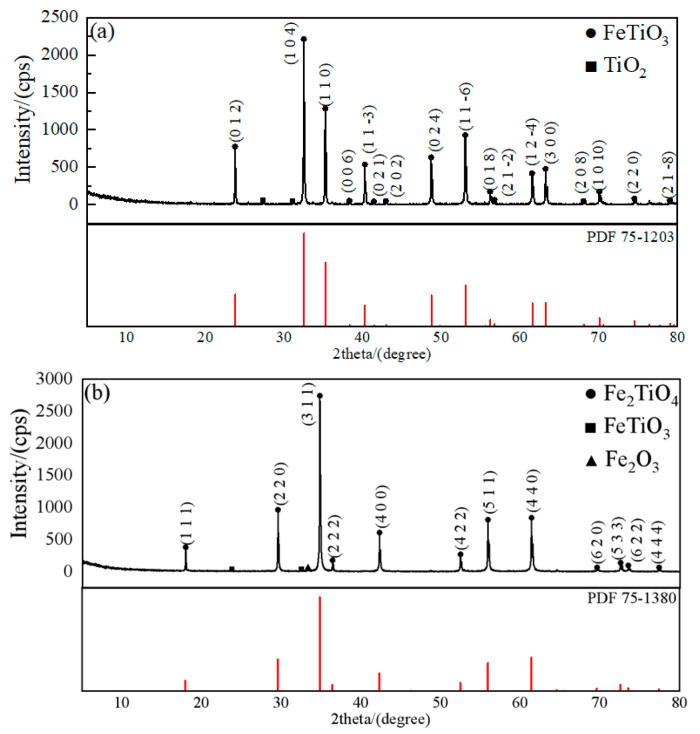
XRD pattern of the sample. (**a**) FeTiO_3_; (**b**) Fe_2_TiO_4_.

As the starting material for diffusion experiments on FeTiO_3_-CaO and Fe_2_TiO_4_-CaO systems, FeTiO_3_ and Fe_2_TiO_4_ were prepared from a 1:1:3 mixture (mole ratio) and 2:2:3 mixture (mole ratio) of Fe (98.0% purity, Tianjin Zhonglian Chemical Reagent Co., Ltd., Tianjin, China), Fe_2_O_3_ (99.0% purity, Shanghai Hushi Laboratory Apparatus Co., Ltd., Shanghai, China), and TiO_2_ (99.0% purity, Xilong Scientific Co., Ltd., Shantou, China) by a mixer for 2 h. The mixed power was placed in a mold with an inner diameter 30 mm and pressed to obtain a mixed sheet body. The mixture was heated at 1150 °C for 10 h in Ar atmosphere. The obtained samples were ground and analyzed by X-ray diffraction (X’Pert PRO MPD/PW3040, Panalytical B.V. Corp., Almelo, The Netherlands). The XRD pattern is displayed in Figure 1. The diffraction peaks of the synthesized FeTiO_3_ and Fe_2_TiO_4_ agree well with the standard reference patterns in JADE (ICDD PDF database)—PDF 75-1203 and PDF 75-1380, respectively. XRD characterization reveals only a few impurity peaks of extremely low intensity, indicating a negligible impurity level and an overall single-phase nature. Accordingly, the synthesized samples exhibit high phase purity, supporting the reliability of the experimental results.

### 2.2. Characterization Methods

Cross-sectional specimens of the reacted diffusion couples, sectioned perpendicular to the reaction layer, were mounted and polished following standard metallographic procedures. During metallographic preparation, the CaO layer exhibited severe pulverization; to facilitate specimen preparation and preservation, excess CaO was gently removed with a soft brush. Pronounced cracks were frequently observed within the FeTiO_3_ and Fe_2_TiO_4_ layers; however, examinations confirmed that the reaction layer was not affected. Surface morphology was examined by scanning electron microscopy equipped with energy-dispersive X-ray spectroscopy (JXA-8100, JEOL Ltd., Beijing, China), and the reaction layer thickness was determined by EDS elemental mapping.

### 2.3. Production of Diffusion Couple

**Figure 2:**
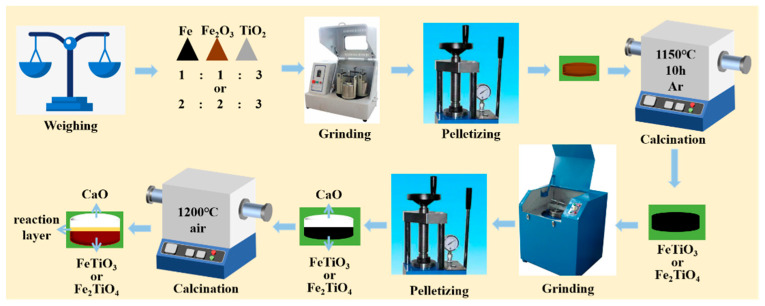
Schematic of the diffusion-couple experimental procedure.

The experimental procedure for a diffusion couple is illustrated in Figure 2. For the preparation of diffusion couples, first, 6 g of FeTiO_3_ (or Fe_2_TiO_4_) powder was loaded into a stainless steel die (inner diameter 30 mm) and uniaxially pressed under an axial load of 800 N for 3 min to obtain a binder-free disk. Without removing this disk from the die, 6g of CaO (98.0% purity, Tianjin ZhiYuan Reagent Co., Ltd., Tianjin, China) powder was added on top and pressed again at 800 N for 3 min to form the CaO layer. Finally, with the two layers remaining in the die, a final compaction at 800 N for 1 min was applied to consolidate the assembly, yielding an intimately bonded FeTiO_3_/Fe_2_TiO_4_–CaO bilayer compact.

### 2.4. Reaction of Diffusion Couple

Guided by thermodynamic calculations, the diffusion behavior was investigated at 1200 °C for 30–120 min, which ensures a clearly measurable reaction while avoiding liquid formation. After preheating the horizontal tube furnace (Luoyang Shenjia Kiln IIndustry Co., Ltd., Luoyang, China) to 1200 °C, the bilayer compact was placed on an alumina crucible holder and inserted into the hot zone so that the reaction proceeded under isothermal conditions. Air was then introduced to initiate the diffusion reaction, with O_2_ and N_2_ flow rates set to 1.05 and 3.95 L/min, respectively. The reaction times were 30, 60, 90, and 120 min.

## 3. Results

### 3.1. Interfacial Microstructure of the Diffusion Couple

**Figure 3:**
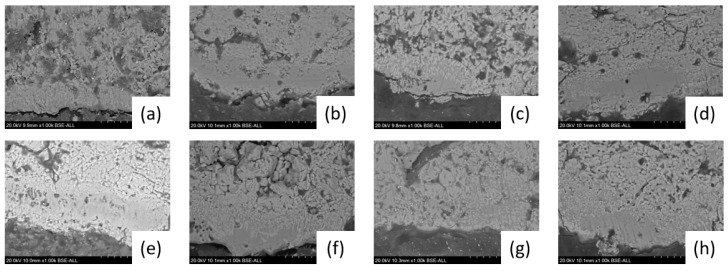
Microstructure of FeTiO_3_-CaO and Fe_2_TiO_4_-CaO diffusion Couples. (**a**) FeTiO_3_ 30 min; (**b**) FeTiO_3_ 60 min; (**c**) FeTiO_3_ 90 min; (**d**) FeTiO_3_ 120 min; (**e**) Fe_2_TiO_4_ 30 min; (**f**) Fe_2_TiO_4_ 60 min; (**g**) Fe_2_TiO_4_ 90 min; (**h**) Fe_2_TiO_4_ 120 min.

Electron probe microanalysis was performed on the FeTiO_3_–CaO and Fe_2_TiO_4_–CaO diffusion couples after the diffusion reaction, and the resulting cross-sectional images are shown in Figure 3. In the interface reaction, the reaction layer is smooth and can be divided into two distinct regions. The region near the CaO matrix exhibits a uniform color, while the portion closer to the FeTiO_3_ and Fe_2_TiO_4_ matrices shows a light-colored needle-like phase. This phenomenon occurs because, with increasing diffusion distance, the concentration of Ca^2+^ decreases, leading to the formation of different reaction products [32].

The cross-sectional images indicate that at 1200 °C, both FeTiO_3_-CaO and Fe_2_TiO_4_-CaO diffusion couples form relatively thick reaction layers within just 30 min. This observation suggests that the rate-limiting step for solid phase mass transfer in the FeTiO_3_-CaO and Fe_2_TiO_4_-CaO systems is not the interface reaction resistance. Another noticeable phenomenon is that the thickness of the reaction layers in both diffusion couples is positively correlated with the sintering time. Moreover, as the sintering time increases, the rate of increase in the reaction layer thickness slows down.

**Figure 4:**
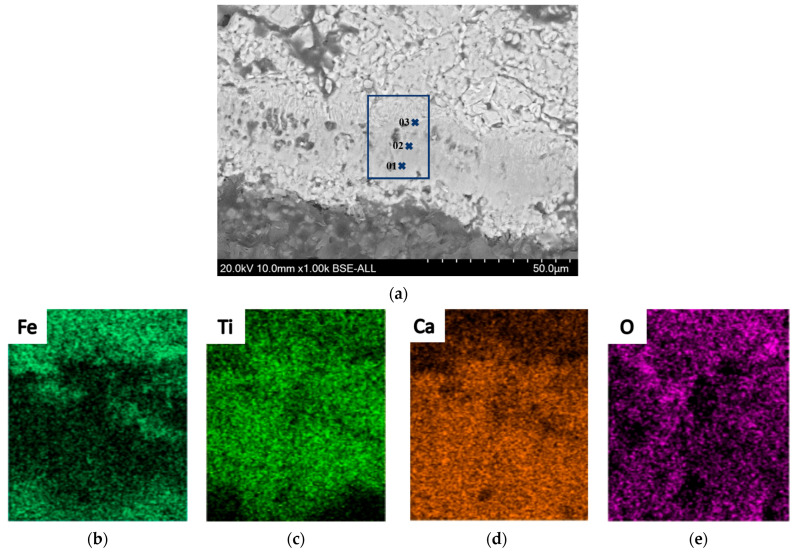
Microstructure and elemental distribution of the Fe_2_TiO_4_-CaO diffusion couple after 30 min of sintering. (**a**) microstructure; (**b**) Fe distribution; (**c**) Ti distribution; (**d**) Ca distribution; (**e**) O distribution.

Both FeTiO_3_ and Fe_2_TiO_4_ decompose into Fe_2_O_3_ and TiO_2_ when heated in air. Therefore, in this study, the interface reaction behavior of the diffusion couple was explained using the example of Fe_2_TiO_4_-CaO sintered for 30 min. Figure 4 shows the EDS-SEM results, with the chemical compositions at different points listed in Table 1. The surface scanning results reveal a distinct titanium-rich region in the reaction layer, and a ferrite-rich region near the CaO matrix. This is because, during the interdiffusion process, Ca^2+^ ions diffuse into the FeTiO_3_ or Fe_2_TiO_4_ matrix, while Fe^3+^ and Ti^4+^ ions also diffuse into the CaO. However, the diffusion of Ti^4+^ requires the substitution of two Ca^2+^ ions to maintain charge neutrality, resulting in a higher electrochemical resistance to diffusion for Ti^4+^ compared to Fe^3+^ [32]. As a result, the diffusion rate of Ti^4+^ is slower than that of Fe^3+^. This conclusion is also supported by the data presented in Table 1.

### 3.2. Interface Formation Mechanisms

In order to gain a better understanding of the phase composition at the diffusion interface, a thermodynamic analysis was conducted by FactSage 8.1. Thermodynamic data were taken from the FactPS and FToxid databases. Reactions (1)–(9) represent the chemical reactions occurring in the FeTiO_3_-CaO and Fe_2_TiO_4_-CaO diffusion couples:

4FeTiO_3_ + O_2_ = 2Fe_2_O_3_ + 4TiO_2_ (1)

2Fe_2_TiO_4_ + O_2_ = 2Fe_2_O_3_ + 2TiO_2_ (2)

TiO_2_ + CaO = CaTiO_3_ (3)

2CaO + Fe_2_O_3_ = Ca_2_Fe_2_O_5_ (4)

CaO + 2Fe_2_O_3_ = CaFe_4_O_7_ (5)

CaO + Fe_2_O_3_ = CaFe_2_O_4_ (6)

CaFe_2_O_4_ + TiO_2_ = CaTiO_3_ + Fe_2_O_3_ (7)

CaFe_4_O_7_ + TiO_2_ = CaTiO_3_ + 2Fe_2_O_3_ (8)

Ca_2_Fe_2_O_5_ + 2TiO_2_ = 2CaTiO_3_ + Fe_2_O_3_ (9)

**Figure 5:**
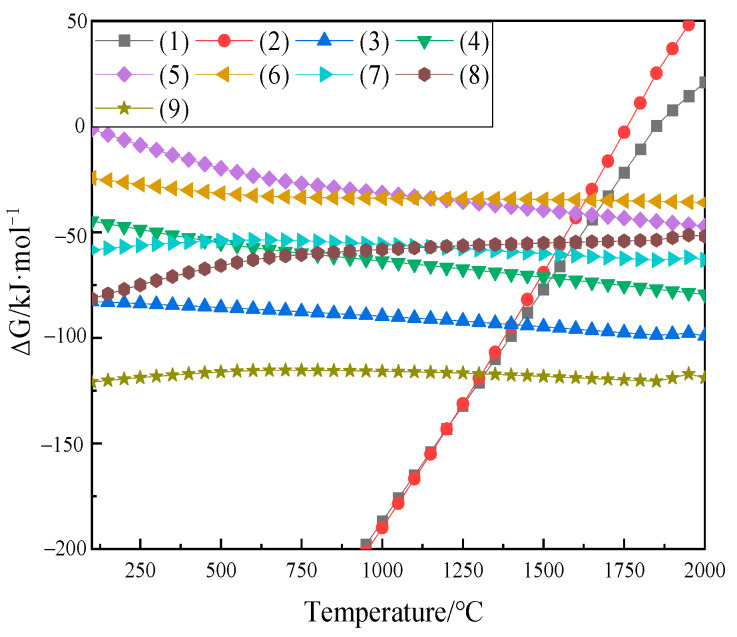
Temperature dependence of ΔG for reactions (1)–(9).

The variation of ΔG for reactions (1)–(9) with temperature is shown in Figure 5. At 1200 °C, FeTiO_3_ and Fe_2_TiO_4_ decompose into Fe_2_O_3_ and TiO_2_. Subsequently, Fe_2_O_3_ reacts with CaO to form calcium ferrite, while TiO_2_ reacts with CaO to form CaTiO_3_. Compared to calcium ferrite, TiO_2_ has a higher affinity for CaO, leading to the formation of CaTiO_3_. This also explains why two different phases are observed in the reaction layer, particularly in regions far from the CaO matrix, as shown in Figure 3.

**Figure 6:**
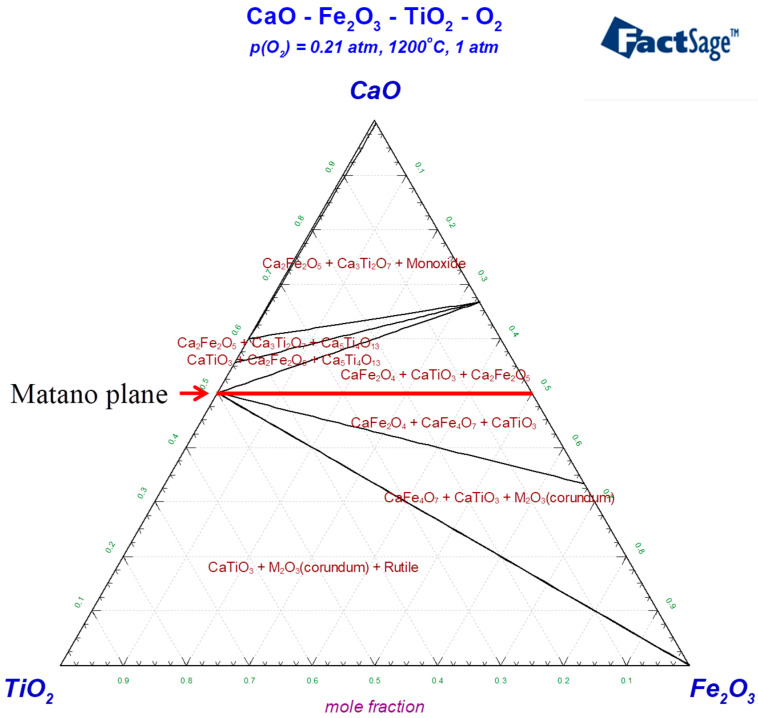
The phase diagram of CaO-TiO_2_-Fe_2_O_3_ at 1200 °C.

The phase diagram of CaO-TiO_2_-Fe_2_O_3_ at 1200 °C is shown in Figure 6, with an oxygen partial pressure of 0.21 atm; the remainder is inert gas. Based on the phase diagram and Gibbs free energy, FeTiO_3_/Fe_2_TiO_4_ decomposes upon heating to form Fe_2_O_3_ and TiO_2_. This study is primarily based on Fick’s second law, and the reaction process was examined using the Matano plane. At the initial contact surface, the molar ratio of FeTiO_3_ to CaO is 1:1, and at the Matano plane, the molar content of CaO is 50%. At the Matano plane, CaO first reacts with the Fe_2_O_3_ and TiO_2_ produced during decomposition to form calcium ferrite and CaTiO_3_. During the diffusion process, the concentration of Ca^2+^ gradually decreases, and the products transform into CaFe_4_O_7_, CaTiO_3_, and Fe_2_O_3_. As the diffusion process continues, the Ca^2+^ concentration in the region farther from the CaO matrix further decreases, causing Ca^2+^ to preferentially react with TiO_2_ to form CaTiO_3_. Due to the depletion of Ca^2+^, the remaining TiO_2_ no longer participates in any further reactions with Fe_2_O_3_.

### 3.3. The Thickness of the Layer of Products

The thickness of the reaction layer was measured as follows. First, a relatively flat, band-like region of the reaction layer in the FeTiO_3_-CaO and Fe_2_TiO_4_-CaO diffusion couples was outlined in CAD 2021 software, with the left and right boundaries kept vertical. The area (S) of the selected region was obtained directly from the CAD calculation and converted to the actual size using the scale bar. The thickness of the reaction layer was then determined by the following equation:

(10) $$S=\int L\;dx=L\bar{x}$$

where L denotes the distance between the left and right boundaries of the reaction layer and $\bar{x}$ represents the average thickness of the reaction layer.

**Figure 7:**
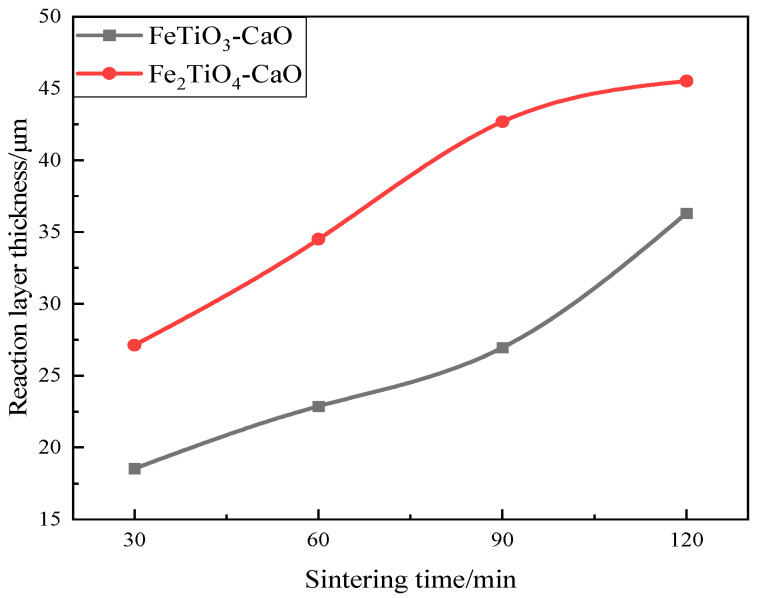
Variation in reaction layer thickness with sintering time.

Figure 7 shows the reaction layer thicknesses of the FeTiO_3_-CaO and Fe_2_TiO_4_-CaO diffusion couples at 1200 °C for different sintering times. A clear positive correlation exists between reaction layer thickness and sintering time, and the layer in the Fe_2_TiO_4_-CaO couple is markedly thicker than that in FeTiO_3_-CaO. The primary difference between the two systems stems from the different concentrations of Fe^3+^ and Ti^4+^ ions generated after thermal decomposition in air. During interdiffusion, substitution of Ca^2+^ sites by Fe^3+^ creates positively charged defects and calcium vacancies; Ti^4+^ produces even more Ca vacancies, but at higher Ti^4+^ concentrations composite defects readily form, blocking diffusion pathways. Moreover, Ti^4+^ exerts a stronger electrostatic attraction on Ca^2+^ than Fe^3+^, thereby hindering Ca^2+^ diffusion. Consequently, under the same sintering time, the Fe_2_TiO_4_-CaO diffusion couple develops a wider reaction–diffusion layer [33].

**Figure 8:**
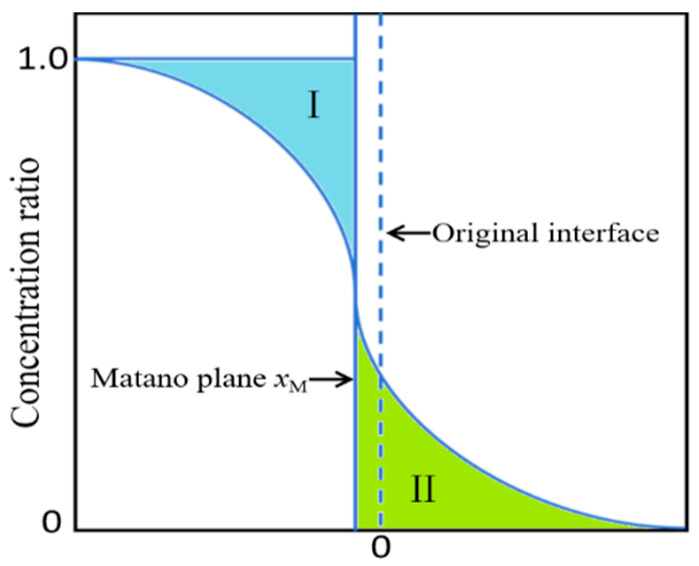
Schematic for determining the Matano plane from the concentration profile.

In a one-dimensional diffusion couple, the Matano plane is determined by the following relation:

(11) $$\int_{-\infty }^{x_{M}}\left(C-C_{1}\right)dx=\int_{x_{M}}^{+\infty }\left(C_{2}-C\right)dx$$

where *C*_1_ and *C*_2_ denote the end member concentrations at the two ends of the couple. The Matano plane was located from the experimental concentration profile using the equal area criterion, as shown in Figure 8, where regions I and II have equal areas.

Intermetallic products formed via cation exchange reactions can be schematically written as ABO_x_ + DO_y_ = ADO_w_ + BO_z_, where A, B, and D are cations and O denotes oxygen. At 1200 °C no liquid phase is generated; hence, layer growth is governed by solid-state diffusion. For one-dimensional diffusion, Fick’s second law applies:

(12) $$\frac{\partial C}{\partial t}=D\frac{\partial^{2}c}{\partial x^{2}}$$

Initial and boundary conditions correspond to two semi-infinite end members brought into contact with the following equations:

(13) $$C=C_{0},\;x<0,\;\;\;t=0$$

(14) C=0, x>0,   t=0

Using the Boltzmann–Matano method, the governing relation can be reformulated. The first step is to locate the Matano plane—the point on the concentration profile that satisfies the mass-balance condition—so that

(15) $$\int_{C_{i}^{+\infty }}^{C_{i}^{0}}xdC_{i}+\int_{C_{i}^{0}}^{C_{i}^{-\infty }}xdC_{i}=0$$

where $C_{i}^{+\infty }$ denotes the terminal composition of the diffusion couple, and $C_{i}^{0}$ denotes the composition at the Matano plane. The temperature dependence of interdiffusion is well described by the Arrhenius relation, implying that *D* increases with *T*:

(16) $$D=D_{0}\cdot \exp(-\frac{Q_{D}}{RT})$$

Here, *D*_0_ denotes the pre-exponential factor, *Q_D_* denotes the activation energy for interdiffusion, *T* denotes the absolute temperature, and *R* denotes the universal gas constant. When growth is limited by diffusion through the product oxide, the mean Gibbs free energy gradient across the layer is given by the following:

(17) $$\frac{dG}{dx}=\frac{G_{1}-G_{2}}{x}$$

Define *G*_1_ and *G*_1_ as the molar free energies of Ca^2+^ at two positions across the layer; the term (*G*_1_ − *G*_2_) is a function of temperature and remains constant under isothermal conditions, and the diffusion flux J is given by the following:

(18) $$J=-D\frac{G_{1}-G_{2}}{x}$$

When growth is diffusion-limited, the thickening rate of the product layer $\frac{dx}{dt}$ proportional to the flux *J*: $\frac{dx}{dt}=\frac{k}{x}$.

Integrating both sides of the equation yields the following:

*x*^2^ = 2*kt* (19)

Thus, *x*^2^ is proportional to *t*, and the reaction layer thickness is obtained from the preceding relation.

The diffusion coefficient *D* is calculated as follows [36]:

*x*^2^ = 4*Dt* (20)

**Figure 9:**
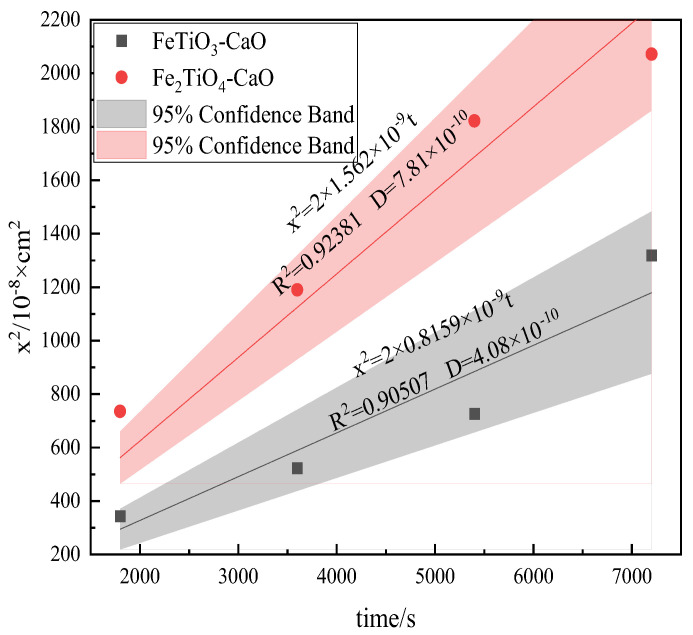
The fitting results between reaction layer thickness and reaction time.

Figure 9 shows the fitting results between reaction layer thickness and reaction time. The interdiffusion coefficients of the FeTiO_3_-CaO and Fe_2_TiO_4_-CaO diffusion couples at 1200 °C are 4.08 × 10^−10^ cm^2^·s^−1^ and 7.81 × 10^−10^ cm^2^·s^−1^, respectively; the reason for this difference has been explained earlier in the text. The regression coefficients (R^2^) are all greater than 0.90, indicating a good fit and lending credibility to the regression equations.

## 4. Conclusions

This study elucidates the interdiffusion behavior of the Fe_2_TiO_4_–CaO and FeTiO_3_–CaO systems during sintering at 1200 °C, thereby informing the selection of basicity in VTM sinter blend design. Such guidance helps suppress the formation of perovskite, leading to improved sinter strength and resistance to reduction degradation. The results also provide a useful reference for phase-selective control in the Fe–Ti–Ca system. The main conclusions are as follows:

(1) Numerous irregular pores form in FeTiO_3_ and Fe_2_TiO_4_ during sintering, weakening the strength of vanadium–titanium sinter. In the FeTiO_3_-CaO and Fe_2_TiO_4_-CaO diffusion couples, differences in the diffusion rates of Ti^4+^ and Fe^3+^ generate distinct Ti-rich and Fe-rich regions, and banded CaTiO_3_ develops adjacent to the FeTiO_3_ and Fe_2_TiO_4_ matrices. This hard, brittle perovskite phase markedly reduces the sinter’s tumble strength.

(2) At 1200 °C, the FeTiO_3_-CaO and Fe_2_TiO_4_-CaO diffusion couples form relatively thick reaction layers within 30 min, indicating that the rate-limiting step is solid-state mass transport rather than the interfacial reaction rate.

(3) At 1200 °C, as Ca^2+^ diffuses into FeTiO_3_ and Fe_2_TiO_4_, CaO first reacts simultaneously with the decomposition products Fe_2_O_3_ and TiO_2_ to form calcium ferrite and CaTiO_3_. With increasing diffusion distance, the Ca^2+^ concentration progressively decreases and the products evolve to CaFe_4_O_7_, CaTiO_3_, and Fe_2_O_3_. As diffusion proceeds further—i.e., in regions farther from the CaO matrix—the Ca^2+^ level drops even more; Ca^2+^ then preferentially combines with TiO_2_ to form CaTiO_3_. Owing to the shortage of Ca^2+^, the remaining TiO_2_ and Fe_2_O_3_ no longer participate in additional reactions.

(4) At 1200 °C, the interdiffusion coefficients of the FeTiO_3_-CaO and Fe_2_TiO_4_-CaO diffusion couples are 4.08 × 10^−10^ cm^2^·s^−1^ and 7.81 × 10^−10^ cm^2^·s^−1^, respectively. The reaction layer thickness can be predicted by the regression equations x^2^ = 2 × 1.562 × 10^−9^ t and x^2^ = 2 × 0.8159 × 10^−9^ t, and the coefficients of determination are all greater than 0.90, indicating reliable fits.

## Figures and Tables

**Table 1:** Elemental compositions (wt%) at the points marked in Figure 4 and corresponding phases.

Point No.	Fe	Ti	Ca	Phase Identified
01	17.1	23.5	31.7	Calcium ferrite and CaTiO_3_
02	38.5	13.6	15.9	CaTiO_3_
03	54.3	9.2	7.8	CaTiO_3_

## Data Availability

The data supporting this study are openly available in FigShare at https://doi.org/10.6084/m9.figshare.29643143.v1.
